# Current role of machine learning and radiogenomics in precision neuro-oncology

**DOI:** 10.37349/etat.2023.00151

**Published:** 2023-07-19

**Authors:** Teresa Perillo, Marco de Giorgi, Umberto Maria Papace, Antonietta Serino, Renato Cuocolo, Andrea Manto

**Affiliations:** University of Campania “L. Vanvitelli”, Italy; ^1^Department of Neuroradiology, “Umberto I” Hospital, 84014 Norcera Inferiore, Italy; ^2^Department of Advanced Biomedical Sciences, University of Naples “Federico II”, 80138 Naples, Italy; ^3^Department of Medicine, Surgery, and Dentistry, University of Salerno, 84084 Fisciano, Italy

**Keywords:** Artificial intelligence, machine learning, radiogenomics, neuro-oncology, glioblastoma, meningioma

## Abstract

In the past few years, artificial intelligence (AI) has been increasingly used to create tools that can enhance workflow in medicine. In particular, neuro-oncology has benefited from the use of AI and especially machine learning (ML) and radiogenomics, which are subfields of AI. ML can be used to develop algorithms that dynamically learn from available medical data in order to automatically do specific tasks. On the other hand, radiogenomics can identify relationships between tumor genetics and imaging features, thus possibly giving new insights into the pathophysiology of tumors. Therefore, ML and radiogenomics could help treatment tailoring, which is crucial in personalized neuro-oncology. The aim of this review is to illustrate current and possible future applications of ML and radiomics in neuro-oncology.

## Introduction

Artificial intelligence (AI) consists of algorithms that are developed to automatically analyze large amounts of data in order to make high-level abstractions [1]. Recently, it has been increasingly used to create tools to enhance medical workflow in multiple fields, though it has proved extremely useful in precision oncology, as it may identify features hidden in the human eye which can guide therapy [2].

**Figure 1:**
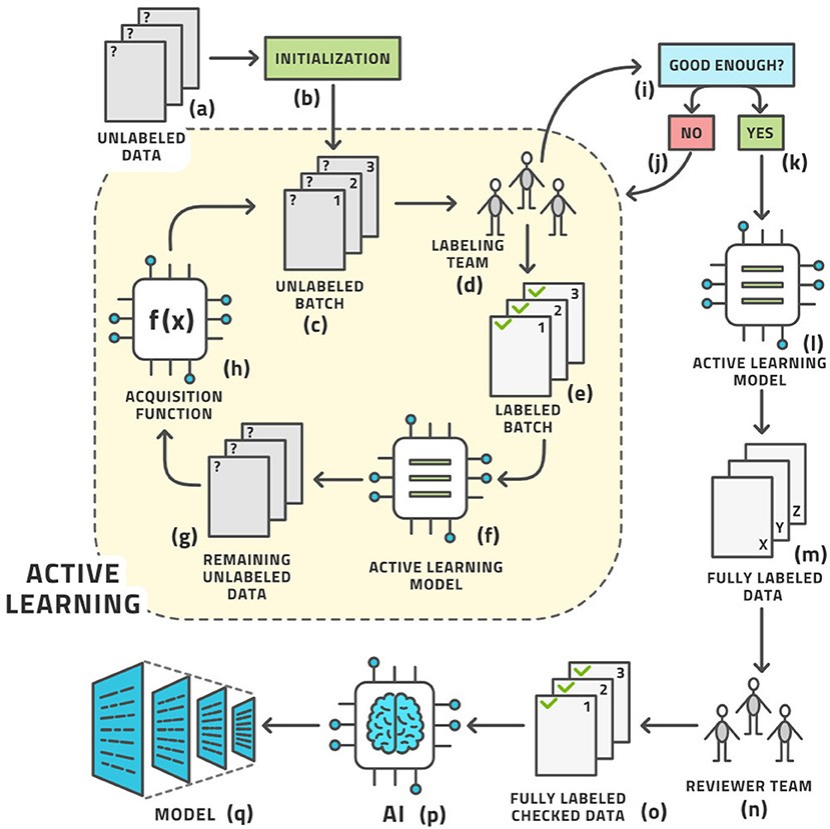
Example of supervised learning *Note.* Reprinted from “Active learning performance in labeling radiology images is 90% effective,” by Bangert P, Moon H, Woo JO, Didari S, Hao H. Front Radiol. 2021;1:748968 (https://www.frontiersin.org/articles/10.3389/fradi.2021.748968/full). CC BY.

**Figure 2:**
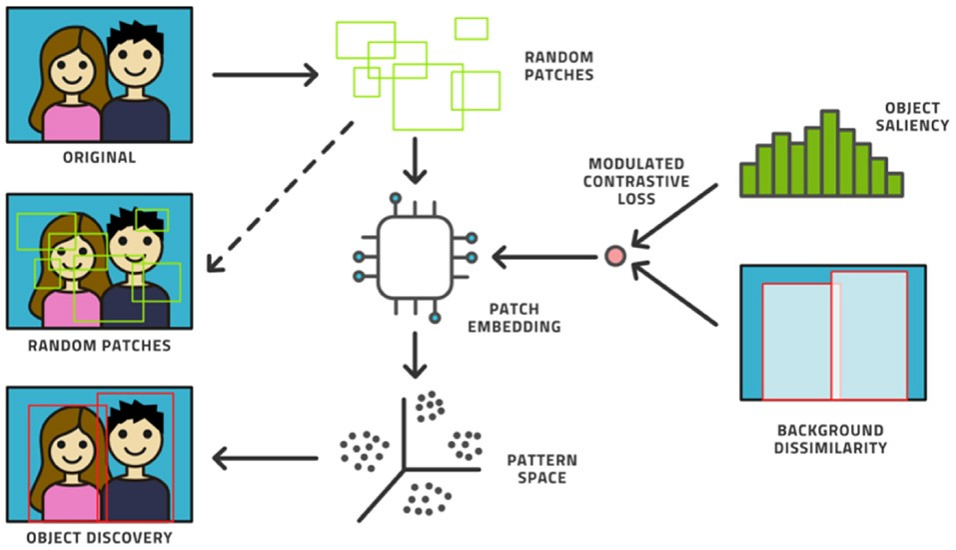
Example of unsupervised learning *Note.* Reprinted from “Active learning performance in labeling radiology images is 90% effective,” by Bangert P, Moon H, Woo JO, Didari S, Hao H. Front Radiol. 2021;1:748968 (https://www.frontiersin.org/articles/10.3389/fradi.2021.748968/full). CC BY.

Machine learning (ML) is a subfield of AI that can be used to automatically analyze large amounts of medical data to solve different problems and it does not require prior explicit programming [3]. As a matter of fact, ML algorithms can learn using different approaches [4]. Supervised (or active) learning is the most used type of learning, and it is based on an external known standard (**Figure 1**). Unsupervised learning automatically identifies hidden structures present in large amounts, without needing an external ground truth (**Figure 2**) [5]. Finally, reinforcement learning uses a trial-and-error process through external positive or negative reinforcement. These different types of learning paradigms may be used in combination [6]. Nowadays, lots of ML algorithms are used in medicine, though radiology has particularly benefited from the use of AI, especially in oncologic patients [7–9]. Deep learning (DL) is a subfield of ML that can be used to analyze large amounts of data in order to make high-level abstractions. The neural network is a subtype of DL that is based on the presence of nodes which are used to create multi-layered networks [10]. The convolutional neural network is a subtype of DL which uses a convolution matrix to extract features from medical images [11].

Radiogenomics, also known as imaging genomics, is a field of AI which can identify relationships between tumor genomics and imaging phenotypes which are not visible by the human eye [12].

Aim of this review is to illustrate the current roles of ML and radiogenomics in precision neuro-oncology.

## Brain gliomas

Brain gliomas (BGs) are the most common primary brain tumors, whose estimated incidence is about 0.2–4.8 cases per 100,000, with peak ages between 45 years and 65 years [13]. The World Health Organization (WHO) distinguishes four histopathological grades, with grade I and II which are defined as low, and III and IV as high grades [14]. Recently, the molecular status of BGs has become crucial to predict prognosis and response to therapy, in particular regarding the mutation status of isocitrate dehydrogenase (IDH), the expression of alpha-thalassemia intellectual disability syndrome X-linked (*ATRX*), the long arm of chromosome 1 (1p)/short arm of chromosome 19 (19q) codeletion, lysine 27-to-methionine (K27M) mutations in the gene histone 3 family 3A (*H3F3A*) and *O*-6-methylguanine-DNA methyltransferase (MGMT) [15].

There are several radiomic features that can be used in BGs. Morphological features are used to characterize the topology of BGs [16]. They are divided into global and local. Global morphological radiomic features evaluate the contour of BGs, considering elements such as perimeters and diameters. On the other hand, local morphological radiomic features evaluate the surface curvature of BGs derived from isosurfaces [17]. Textural radiomics may be of different kinds. Structural methods are used to identify patterns in medical images which cannot be seen by the human eye, and Gabor descriptors are among the most used filters of this group [16]. Statistical texture radiomics methods, such as histograms of oriented gradients and grey-level co-occurrence matrix, analyze the distribution of grey values and local features [18]. Functional radiomics is built to directly address specific pathophysiological properties of tissues [16]. For instance, in neuro-oncology, angiogenesis is frequently evaluated by this method, as it affects treatment response [19]. Finally, semantic features of BG such as edema, contrast enhancement, and necrosis can predict survival [20].

Radiogenomics may be extremely useful in BGs, as it can be used to predict the molecular status of the tumor in the pre-surgical setting [21]. For instance, Gutman et al. [22] used radiogenomics to analyze magnetic resonance (MR) exams of glioblastoma multiforme (GM) before neurosurgery, and it was able to identify correlations between imaging characteristics of the tumors and their genetic expression. Similarly, Zinn et al. [23] used radiogenomics to identify MR features of GM associated with the expression of specific types of microRNAs (miRNAs, for example, the mesenchymal type of GM shows a low expression of miR-219). Finally, Li et al. [24] used radiogenomics to identify MR features that could predict MGMT methylation status in GM, with good accuracy [area under the curve (AUC) of 88%]. On the other hand, Qian et al. [25] used radiomics and radiogenomics to analyze MR images of low-grade BGs in order to identify imaging features associated with hypoxia, angiogenesis, apoptosis, and cell proliferation with good correlation with prognosis.

BGs are an extremely heterogenous group of tumors in terms of gene expression and clinical outcomes, thus prediction of grading before neurosurgery is challenging [26]. In this setting, AI has proved to be useful as it can identify imaging features that are not detectable by the human eye [27]. For example, Takahashi et al. [28] created an ML-based tool which could automatically extract hidden features from MR of brain metastasis (BM, in particular using apparent diffusion coefficient maps), which accurately predicts the grading of BGs. ML may also be used to distinguish low to high-grade BGs. Skogen et al. [29] used a dataset of 95 MR of BGs and ML automatically differentiated low from high-grade BM, with high sensitivity and specificity (93% and 81%, respectively). Similarly, Tian et al. [30] trained an ML tool on a dataset of multiparametric MR images of BGs in order to automatically differentiate low from high-grade BGs and it showed high accuracy (> 96%).

AI has also proved to be useful in the prediction of the prognosis of BGs, especially in high-grade tumors. In this setting, one of the possible applications of ML is the ability to predict the aggressiveness of BGs, thus guiding treatment before neurosurgery [31]. Hypoxia plays a crucial role in BGs, as it involved tumor neovascularization, propensity to invasiveness, and resistance to treatment [32]. Beig et al. [33] demonstrated that ML can be used to identify features present in pre-treatment MR of GM that correlate with the extent of hypoxia. On the other hand, the morphology of tumors may be used to predict the prognosis of BGs. For example, Prasanna et al. [34] evaluated the mass effects induced by GM using enhance MR, showing that this feature can be analyzed to predict survival.

## BM

BM is a frequent finding as it is presented in almost 20% of cases of patients that have cancer involving anatomic sites other than the nervous system [35]. In this setting, ML and radiogenomics can be used to identify the primary tumor, evaluate the mutation status and aggression of BM, and predict response to treatment and risk of recurrence.

ML and radiogenomics may be extremely helpful in the setting of the identification of unknown primary tumors. For instance, Kniep et al. [36] retrospectively studied 189 patients with primary breast cancer, lung cancer, gastric cancer, and melanoma who developed BM and analyzed enhanced and non-enhanced T1 and fluid-attenuated inversion-recovery (FLAIR) images using an ML-based algorithm in order to identify the primary tumor, with high AUC (between 64–82% depending on the tumor).

Prediction of mutation status in BM may help guide treatment [37]. Ahn et al. [38] demonstrated that ML can be used to predict glomerular filtration rate (GFR) mutation status in BM from lung cancer using enhanced MR, with high accuracy (AUC of 86.81%). Similarly, Park et al. [39] used ML to extract features from MR (in particular diffusion tensor maps and enhanced T1 images) to identify the epidermal growth factor receptor (*EGFR*) mutation status of BM from non-small cell lung cancer, with an AUC of 73%. Chen et al. [40] used enhanced T1, T2, and FLAIR images to predict the mutation on *EGFR*, anaplastic lymphoma kinase (*ALK*), and Kirsten rat sarcoma viral oncogene homologue (*KRAS*) in BM from patients diagnosed with primary lung cancer, verified by genotype testing. The model on *EGFR*, *ALK*, and *KRAS* incorporating both radiomics and clinical information resulted in AUC values of 91.2%, 91.5%, and 98.5% respectively.

ML and radiogenomics may also have a crucial role in the prediction of treatment and progression, especially when patients are eligible for radiation and chemotherapy. For example, Prasanna et al. [41] developed an ML feature called COLLAGE which could distinguish recurrence from relapse in patients with BM after radiotherapy. Similarly, Huang et al. [42] created an ML tool to identify prognostic factors in BM treated with Gamma Knife radiosurgery in patients with non-small cell lung cancer.

Finally, Peng et al. [43] used ML and radiomics to distinguish true progression from radionecrosis after stereotactic radiation therapy for BM using 51 radiomic features extracted from MR images. It showed high sensitivity and specificity (65.38% and 86.67%, respectively).

## Meningioma

Meningiomas are the most frequent tumors of the meninges and although they are frequently low-grade, it is possible to identify 15 pathological subtypes, some of which may be aggressive [44]. Therefore, ML and radiogenomics may be used to identify features in the presurgical setting that are associated with aggressiveness, thus guiding tailored therapy [45].

Yan et al. [46] proved that ML analysis (in particular of texture and shape of tumors) of MR before neurosurgery can predict the grading meningioma, in particular of those of grade II. Similarly, Zhu et al. [47] used DL to develop a model to predict meningioma grading non-invasively. Finally, Hamerla et al. [48] used multiparametric MR imaging (MRI) from different centers to create an ML-based algorithm, which proved could automatically distinguish low from high-grade meningioma.

ML can also be used to identify morphologic features associated with prognosis. For instance, low sphericity correlated with local recurrence and less favorable overall survival [49].

ML can also be used to differentiate meningioma subtypes. In particular, Niu et al. [50] used 385 radiomics features extracted from medical images in the pre-surgical setting, obtaining satisfactory performance.

Radiomics can also be used to predict brain invasion in meningiomas. In particular, Zhang et al. [51] created a model incorporating radiomic and clinical features, which showed great performance and high sensitivity.

## Pediatric brain tumors

Pediatric brain tumors are the most common solid tumors in children, and MR is the preferred imaging modality for diagnosis and staging [52]. Regardless of the ongoing progresses in MR, invasive diagnosis of pediatric brain tumors is crucial in clinical practice and radiomics could be used for non-invasive characterization of brain tumors, thus guiding tailored treatment [37].

Medulloblastoma is a highly malignant pediatric brain tumor, which can be classified into four different groups, which are wingless-type MMTV integration site family (*WNT*), sonic hedgehog (*SHH*), group 3, and group 4 [53]. Even though no single imaging characteristic is pathognomonic of any precise subgroup, some imaging features are significantly more frequent in one subgroup compared to others and might even be extremely precise for a peculiar molecular subgroup. These include a lateralized cerebellar location for the SHH-subgroup, cerebellopontine angle location for the WNT-subgroup, and inferior location with dilation of superior recess of the fourth ventricle for group 4 tumors [54]. As there are subgroup-specific imaging characteristics, researchers have created ML models for the pre-operative prediction of molecular subgroups. For instance, Chang et al. [55] analyzed MR radiomics features to find the imaging surrogates of the 4 molecular subgroups of medulloblastoma. A total of 253 MR radiomic features were generated from each subject for comparison between different molecular subgroups with 8 radiomics features that were significantly different between the 4 molecular subgroups. Pre-operative identification of the molecular subgroup of medulloblastoma is extremely important as the extent of resection depends on the molecular subtype [56]. Therefore, Thompson et al. [57] analyzed the prognostic value of the extent of resection in a retrospective multi-institutional cohort involving 787 patients with medulloblastoma. Only in group 4 tumors, wide neurosurgical resection was associated with a significant increase in progression-free survival, but not in overall survival. Regarding prognosis prediction, it is important to identify cerebral spinal fluid dissemination. In this setting, ML has been used to enhance the identification of spinal fluid dissemination of disease using preoperative-enhanced T1 images in children with medulloblastoma [58]. The combined model incorporating clinical and radiomic features had the best predictive performance in the training cohort with an AUC of 89%.

Pediatric low-grade gliomas are the most common brain tumors in children and comprise a heterogeneous variety of tumors classified by the WHO as grades I or II. Molecular characterization of sporadic pediatric low-grade gliomas has identified frequent alterations in the mitogen-activated protein kinas pathway, most commonly fusions or mutations in the B-raf proto-oncogene (*BRAF*) gene. The 2 major *BRAF* gene alterations are *BRAF* fusion and *BRAF V600E* point mutation (p.V600E) [59]. In a retrospective study, radiomics-based prediction of *BRAF* status in pediatric low-grade gliomas appears feasible. In particular, Wagner et al. [60] used ML to analyze FLAIR MR images of 115 pediatric patients with low-grade gliomas. The ML tool predicted *BRAF* status with an AUC of 75% on the internal validation cohort (AUC for the external validation was 85%).

Diffuse intrinsic pontine gliomas (DIPGs) are brain tumors that predominantly affect children and have dismal survival [61]. An international study demonstrated that an ML model was useful for the prediction of prognosis in the setting of DIPG, outperforming the clinical-only model [62]. Adding clinical features to radiomics slightly improved performance.

ML may also help in the differential diagnosis of pediatric posterior fossa tumors such as medulloblastoma, ependymoma, and astrocytoma. Their prognosis and therapy are different because of the variety in molecular subtyping. Therefore, early and correct diagnosis is important to guide treatment. In a retrospective study, Wang et al. [63] investigated a non-invasive MR-based ML algorithm to classify the histologic tumor types of pediatric posterior fossa brain tumors. They used MR images before surgery of 99 patients histologically confirmed (59 medulloblastomas, 13 ependymomas, and 27 astrocytomas). In particular, the apparent diffusion coefficient proved to be more useful than T1 and T2 images in differentiating pediatric posterior fossa brain tumors.

## Limitations

Although ML and radiogenomics have shown multiple promising applications in neuro-oncology, there are several limitations that must be considered.

Firstly, the reproducibility of ML and radiogenomics in multicenter studies is still poor due to different acquisition parameters and the use of non-standard pipelines. A possible solution is the use of an eternal dataset to give an unbiased estimation of generalization error [63]. Plus, several online platforms have been developed to offer a standardized ML pipeline [64, 65]. Finally, several guidelines have been developed to standardize the development of ML and radiogenomics [66].

Segmentation is an important step in most of the training processes of ML algorithms. This task, which is frequently done manually, maybe a source of bias due to inter-observer variability [67]. Automatic segmentation may be a possible solution, although the software is frequently different among centers. Furthermore, some tumors may have insufficient anatomical contrast which may affect segmentation [53].

Finally, most published scientific studies about ML and radiogenomics in neuro-oncology have a low level of evidence due to a lack of pre-processing, open-source code, and data used during the study and test-retest study [66].

## Conclusions

ML and radiogenomics have shown to be useful in multiple subfields of neuro-oncology. In particular, it could predict tumor genetics and possibly give new insights into the pathophysiology of brain tumors. Furthermore, it could guide treatment choices, leading to personalized neuro-oncology.

## Abbreviations

AI: artificial intelligence
AUC: area under the curve
BGs: brain gliomas
BM: brain metastasis
*BRAF*: B-raf proto-oncogene
DL: deep learning
*EGFR*: epidermal growth factor receptor
FLAIR: fluid-attenuated inversion-recovery
GM: glioblastoma multiforme
ML: machine learning
MR: magnetic resonance

## Declarations

### Copyright

© The Author(s) 2023.
